# Canakinumab in Yao Syndrome: Insights From a Comprehensive Case Report and Literature Review 

**DOI:** 10.7759/cureus.62245

**Published:** 2024-06-12

**Authors:** Anam Ahmad, Adam Kilian

**Affiliations:** 1 Rheumatology, Saint Louis University School of Medicine, St. Louis, USA; 2 Rheumatology, St. Luke's Hospital, Chesterfield, USA

**Keywords:** interleukin-1, periodic fever syndrome, canakinumab, nod2, yao syndrome

## Abstract

Yao syndrome, a rare autoinflammatory disorder linked to mutations in the nucleotide-binding oligomerization domain-containing protein-2 (NOD2) gene, manifests through periodic fever, polyarthritis, dermatitis, gastrointestinal disturbances, and sicca-like symptoms. The therapeutic landscape is limited, primarily encompassing glucocorticoids, interleukin-1 (IL-1), and IL-6 inhibitors. This report details the case of a teenager with periodic fevers, arthritis, livedo reticularis, and NOD2 gene mutations R702W and IVS8+158C consistent with Yao syndrome. The individual demonstrated significant improvement with canakinumab therapy. This case report aims to enhance recognition and understanding of Yao syndrome's clinical spectrum and management options.

## Introduction

Yao syndrome, identified in 2011 by Yao et al. and previously known as nucleotide-binding oligomerization domain-containing protein 2 (NOD2)-associated autoinflammatory disease, is an autoinflammatory disease characterized by periodic fevers and abnormal inflammation affecting multiple body systems, particularly the skin, joints, and gastrointestinal system, driven by dysregulation within the innate immune system [1]. With a global prevalence estimated between 1 and 10 cases per 100,000 adults, Yao syndrome often manifests around the median age of 33 and exhibits a female predominance at a ratio of 2:1 [2]. While typically sporadic, up to 15% of cases demonstrate familial clustering [3]. Yao syndrome is associated with specific mutations in the NOD2 gene located on chromosome 16, including notably NOD2 IVS8+158 and R702W [3].

## Case presentation

A 19-year-old female presented with a two-month history of recurring episodes of polyarthralgia, myalgias, and fevers peaking at 103 °F, each accompanied by night sweats and lasting several days before subsiding. Episodes of fever were interspersed with symptom-free intervals but occurred frequently enough to significantly impact her quality of life. The patient's articular symptoms were extensive, involving the neck, back, knees, hands, and feet, characterized by morning stiffness exceeding 45 minutes, reduced range of motion, and notable swelling in the lower extremities. Concurrently, she experienced nausea, vomiting, and a 20-pound unintentional weight loss. Her family history was notable for a father with Crohn's disease and a mother with seronegative rheumatoid arthritis and Sjögren's disease. Physical examination conducted during an inter-episodic period revealed livedo reticularis but was otherwise unremarkable. Initial laboratory studies are included in Table 1, revealing leukocytosis, and elevated inflammatory markers, infectious and hematological workup was negative.

**Table 1:** Initial laboratory investigations.

Laboratory tests	Values	Reference range and units
WBC	13.6 x 10^3^	3.5-10.5 x 10^3^/uL
Hemoglobin (Hb)	12.6	11.9-15.8 g/dL
Platelets	343 x 10^3^	150-400 x 10^3^/uL
Creatinine	0.94	0.56-0.96 mg/dL
Aspartate aminotransferase (AST )	15	5-34 units (U)/L
Alanine aminotransferase (ALT)	40	5-55 U/L
Erythrocyte sedimentation rate (ESR)	38	0-20 mm/hour
C-reactive protein (CRP )	9.43	<0.5 U/L
Creatinine kinase (CK)	19	30-200 U/L
Aldolase	10.0	1.2-7.6 U/L
Antinuclear antibody (ANA)	Negative	Negative
Extractable nuclear antigen (ENA) profile	Negative	Negative
Rheumatoid factor (RF)	Negative	Negative
Anti-cyclic citrullinated peptide (CCP)	Negative	Negative
Myositis panel	Negative	Negative
Antineutrophil cytoplasmic antibodies (ANCA) panel	Negative	Negative
Complement 3	165	82-193 mg/dL
Complement 4	30	15-57 mg/dL
Immunoglobin G	797	767-1,590 mg/dL
Human leukocyte antigen (HLA)-B27	Negative	Negative

**Figure 1:**
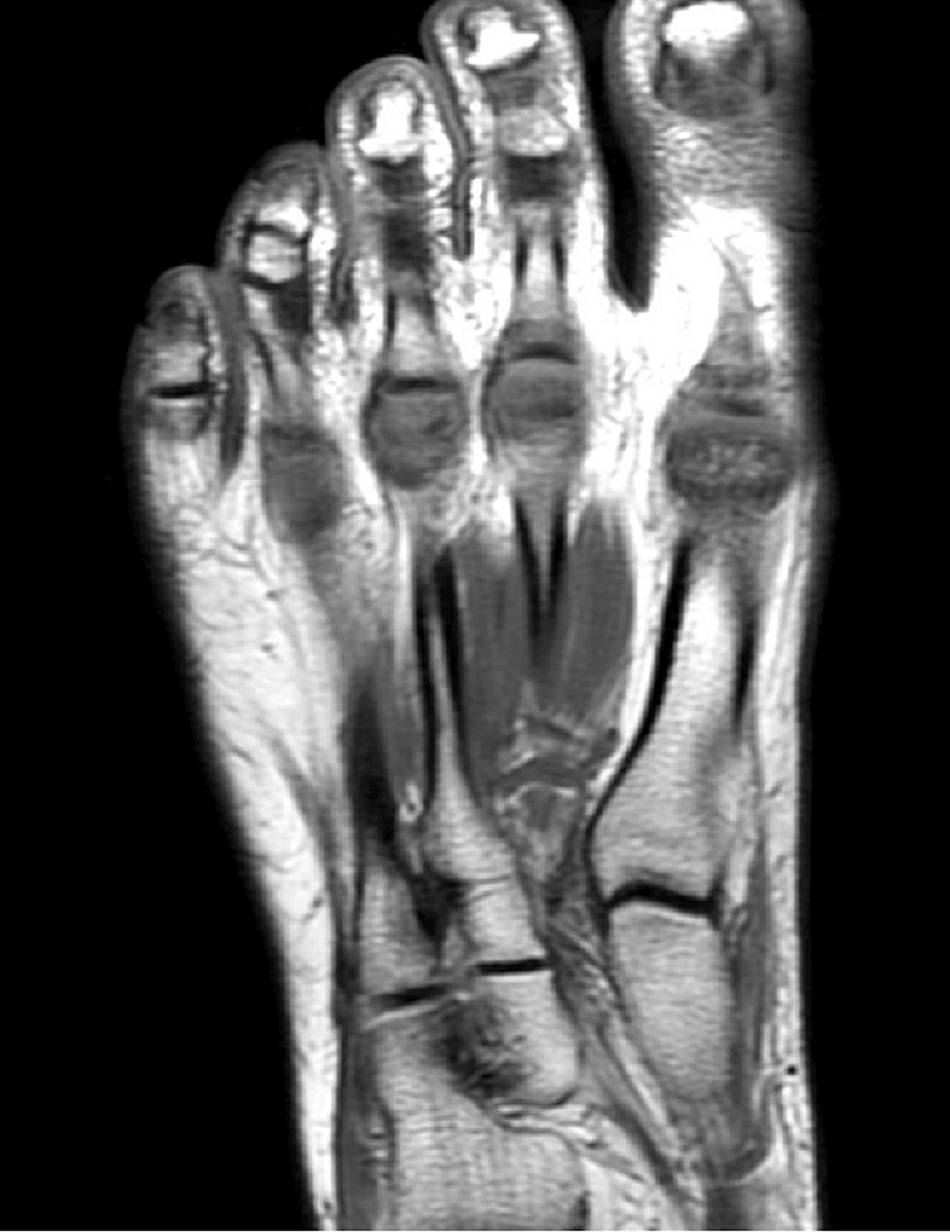
Magnetic resonance imaging of the right foot revealing an edema-like marrow signal within the heads of the second through fifth metatarsals with associated effusions or enhancing synovitis.

Laboratory and imaging evaluations, including bone marrow and lymph node biopsies, ruled out infectious and malignant etiologies. Initial treatment with steroids provided temporary relief; however, her symptoms recurred post-taper. Subsequent treatment with naproxen 500 mg twice daily offered only minimal relief. Reassessment of her arthralgias by an alternative rheumatologist and a magnetic resonance imaging (MRI) of the right foot identified synovitis of metatarsophalangeal joints, leading to a diagnosis of seronegative rheumatoid arthritis and initiation of methotrexate therapy which provided minimal relief (Figure 1). A comprehensive genetic panel confirmed the presence of NOD2 mutations R702W and IVS8+158C. Diagnosis of Yao syndrome was established, leading to effective management with canakinumab 150 mg administered subcutaneously every four weeks, resulting in symptom resolution.

## Discussion

Yao syndrome is an autoinflammatory disease characterized by periodic fevers and abnormal inflammation affecting multiple body systems, particularly the skin, joints, and gastrointestinal system. The fevers are typically severe, lasting several days, and can occur with intervals ranging from weeks to months [4]. Approximately 90% of patients experience skin manifestations, presenting as erythematous plaques and patches that may persist for days to weeks, often histologically identified as spongiotic dermatitis [5]. Arthritis, which occurs in about 80% of affected individuals, can vary from monoarticular to polyarticular forms, contributing significantly to the disease burden. Yao syndrome commonly involves gastrointestinal symptoms like abdominal pain and bloating, sicca complex, and other systemic manifestations including weight loss and pleurisy associated with pericarditis or pleuritis [3,4]. The diverse clinical spectrum of Yao syndrome necessitates a thorough diagnostic approach that relies on both clinical and genetic evaluations. 

Diagnosis of Yao syndrome is contingent upon the recurrence of specific symptoms, the identification of characteristic genetic mutations, and the exclusion of other autoinflammatory disorders [6]. The diagnostic criteria typically require two major and one minor clinical feature, along with confirmatory molecular findings as described in Table 2. Central to the genetic underpinnings of Yao syndrome are mutations in the NOD2 gene. This gene encodes a cytosolic protein that recognizes bacterial muramyl dipeptide, playing a pivotal role in the innate immune response [7]. It is expressed in macrophages, monocytes, and dendritic cells, facilitating the initiation of immune responses essential for bacterial clearance [7]. Mutations in NOD2 have been implicated in Yao syndrome, inflammatory bowel diseases, and Blau syndrome [6,7]. The majority of patients with Yao syndrome exhibit the NOD2 IVS8+158 mutation, and approximately 30% also carry the concurrent R702W variation. These genetic markers were notably present in our patient, solidifying the diagnosis in conjunction with her clinical symptoms [6]. 

Once diagnosed, the treatment of Yao syndrome is tailored to the severity and phenotype of the disease. Patients typically do not respond well to colchicine, which is effective in other periodic fever syndromes. In mild cases, glucocorticoids and sulfasalazine may be sufficient [8]. However, for severe or refractory disease, inhibitors of interleukin-1 (IL-1) or IL-6 can be used [9,10]. Our patient demonstrated significant improvement with canakinumab, an IL-1 inhibitor, administered at 150 mg subcutaneously every four weeks, highlighting its efficacy in managing severe inflammatory responses associated with this syndrome.

**Table 2:** Diagnostic criteria of Yao syndrome.

Clinical criteria
Major: Periodic recurrence of symptoms more than twice; recurrent fever or dermatitis or both
Minor: Inflammatory arthritis or distal extremity swelling; abdominal pain or diarrhea/both; sicca symptoms; pericarditis or pleuritis or both
Molecular criteria
NOD2 IVS8+158 and/or R702W
Exclusion criteria
Systemic lupus erythematosus, vasculitis, inflammatory bowel disease, Blau syndrome, adult sarcoidosis, and primary Sjögren’s syndrome

## Conclusions

Yao syndrome is a rare autoinflammatory disorder characterized by distinct phenotypic and genotypic traits. The complexity of this condition underscores the importance of a comprehensive approach to diagnosis and treatment, integrating both clinical insights and molecular genetic analysis. Prompt recognition of its diverse features can prevent diagnostic delays and facilitate earlier intervention, potentially improving patient outcomes. This case underscores the critical need for increased awareness and knowledge dissemination among clinicians to ensure timely diagnosis and effective management of Yao syndrome.
